# Effects of *Mentha suaveolens* Essential Oil on *Chlamydia trachomatis*

**DOI:** 10.1155/2015/508071

**Published:** 2015-01-22

**Authors:** Rosa Sessa, Marisa Di Pietro, Fiorenzo De Santis, Simone Filardo, Rino Ragno, Letizia Angiolella

**Affiliations:** ^1^Department of Public Health and Infectious Diseases, “Sapienza” University, Piazzale Aldo Moro 5, 00185 Rome, Italy; ^2^Department of Drugs Chemistry and Technology, “Sapienza” University, Piazzale Aldo Moro 5, 00185 Rome, Italy

## Abstract

*Chlamydia trachomatis*, the most common cause of sexually transmitted bacterial infection worldwide, has a unique biphasic developmental cycle alternating between the infectious elementary body and the replicative reticulate body. *C. trachomatis* is responsible for severe reproductive complications including pelvic inflammatory disease, ectopic pregnancy, and obstructive infertility. The aim of our study was to evaluate whether *Mentha suaveolens* essential oil (EOMS) can be considered as a promising candidate for preventing *C. trachomatis* infection. Specifically, we investigated the *in vitro* effects of EOMS towards *C. trachomatis* analysing the different phases of chlamydial developmental cycle. Our results demonstrated that EOMS was effective towards *C. trachomatis*, whereby it not only inactivated infectious elementary bodies but also inhibited chlamydial replication. Our study also revealed the effectiveness of EOMS, in combination with erythromycin, towards *C. trachomatis* with a substantial reduction in the minimum effect dose of antibiotic. In conclusion, EOMS treatment may represent a preventative strategy since it may reduce *C. trachomatis* transmission in the population and, thereby, reduce the number of new chlamydial infections and risk of developing of severe sequelae.

## 1. Introduction

*Chlamydia trachomatis*, an obligate intracellular pathogen, is the leading cause of bacterial sexually transmitted diseases in the world with an estimated over 105 million new cases every year [1]. It manifests as cervicitis, salpingitis, and endometritis and can be transmitted to infants during delivery resulting in neonatal conjunctivitis and pneumonitis [2]. Chlamydial genital infections have also been reported to increase human immunodeficiency virus (HIV) transmission [2, 3] and influence the development of human papilloma virus induced adenocarcinoma [2, 4].

A major concern with chlamydial genital infections is that approximately 80% of women are asymptomatic, thus resulting in a reservoir for onwards transmission in the population. Consequently,* C. trachomatis* untreated infections can progress leading to chronic severe sequelae, including pelvic inflammatory disease, ectopic pregnancy, and obstructive infertility [2].

Similarly to* C. trachomatis*,* Chlamydia pneumoniae* has also been associated with several chronic diseases such as atherosclerosis, Alzheimer's disease, asthma, and more recently osteoporosis-related bone loss [5–7].

Several studies have suggested that the development of* Chlamydiae* associated chronic diseases may be related to the persistent form that can arise under stressful growth conditions imposed, for example, by antibiotic treatment and herpes simplex virus type 2 (HSV-2) coinfection [8, 9].

*C. trachomatis* has an intriguing and unique biphasic developmental cycle alternating between the extracellular, infectious, metabolically inactive elementary body (EB) and the intracellular, noninfectious, metabolically active reticulate body (RB). Apart from EB and RB, a third nonreplicating and noninfectious form, called persistent form, has been described to be responsible for establishing chronic infections since they are able to evade the host immune response and are more difficult to eradicate by antibiotics [8].

The existence of* C. trachomatis* EBs and RBs as well as the persistent forms makes the treatment of infection a challenge. Beta-lactam antibiotics, commonly used against bacterial infections because they are able to inhibit the peptidoglycan synthesis, are ineffective towards* C. trachomatis* for the lack of a classical peptidoglycan [10], and some of them such as penicillin G are even able to induce persistent forms [8, 9].

In contrast, macrolides including azithromycin and erythromycin appear to be effective against* C. trachomatis* [11] although several clinical treatment failures have been reported [12, 13]. The major cause for* C. trachomatis* therapeutic failure may be chlamydial resistance to antibiotic [14–16]. In addition, the phenomenon of antibiotic resistance in* C. trachomatis* could be more complex since it may be also related to the development of persistent forms [17].

Thus, given the impact of asymptomatic chlamydial infections on reproductive outcomes as well as the emerging resistance of* C. trachomatis* to antibiotics and the risk of developing persistent forms, preventing new infections becomes a priority.

Recently, a great interest in natural products as alternative treatment for preventing infections has been raised in view of their extensive biological effects including anticancer, antioxidant, and other pharmacological activities [18]. Essential oils derived from aromatic medicinal plants, such as* Mentha* species, have been explored and gained prominence; in previous studies, it has been demonstrated that* Mentha suaveolens* essential oil was effective against yeasts, fungi, and viruses [19–24].

Therefore, the aim of our study was to evaluate whether* Mentha suaveolens* essential oil can be considered as a promising candidate for preventing* C. trachomatis* genital infections. Specifically, we investigated the* in vitro* effects of* Mentha suaveolens* essential oil on the lymphogranuloma venereum strain of* C. trachomatis* analysing the different phases of chlamydial developmental cycle.

## 2. Materials and Methods

### 2.1. Essential Oil

*Mentha suaveolens* essential oil (EOMS) was obtained from wild-type plants grown in Tarquinia forests (Rome, Italy) and was extracted by 4 h hydrodistillation of the aerial parts using a Clevenger-type apparatus as previously described by Angiolella et al. [21].

### 2.2. Propagation and Titration of* C. trachomatis*

*C. trachomatis* lymphogranuloma venereum (LGV) serovar L2 (ATCC-VR-902B) was propagated in HeLa cells (ATCC CCL-2) grown in 6-well plates (1 × 10^6^ cells/well) in Dulbecco's Modified Eagle's medium (DMEM, Euroclone, Italy) high glucose supplemented with 10% heat inactivated foetal calf serum (FCS, Euroclone, Italy). Infected HeLa cells were centrifuged at 900 ×g for 60 min at 37°C. The supernatant was removed and replaced with DMEM high glucose supplemented with 10% FCS, 10 mM HEPES (Sigma-Aldrich, St. Louis, USA), and 1 *μ*g/mL cycloheximide (Sigma-Aldrich, St. Louis, USA). After 48 h of incubation at 37°C and 5% CO_2_, infected cells were harvested in sucrose-phosphate-glutamate buffer (SPG buffer) (0.2 M sucrose, 3.8 mM KH_2_PO4, 6.7 mM Na_2_HPO4, and 5.5 mM glutamic acid at pH 7.4) and vortexed with sterile glass beads for 2–5 min. After removal of cell debris by centrifugation at 250 ×g for 10 min, the supernatant was centrifugated at 20,000 ×g and 4°C for 20 min and the pellet, resuspended in SPG buffer, was stored at −70°C.

The infectious titre (inclusion-forming units (IFU)/mL) was assessed by immunofluorescence assay. Briefly, HeLa cells (2.0 × 10^5^ cells/well), grown on glass coverslips in 24-well plates, were infected with tenfold serial dilutions of chlamydial EBs suspension, incubated for 48 h at 37°C and 5% CO_2_, fixed with methanol, and stained with fluorescein isothiocyanate conjugated monoclonal (FITC) antibody against* C. trachomatis* MOMP (Micro-Trak* Chlamydia trachomatis* Culture Confirmation Test, Trinity Biotech, USA). The total number of IFUs was enumerated by counting all microscope fields using a fluorescence microscope (400x magnification).

### 2.3. Cytotoxicity Assay

The cytotoxicity of EOMS was determined on HeLa cells using the MTT (methylthiazolyldipheniltetrazolium bromide) assay. Briefly, 1.0 × 10^4^ HeLa cells were seeded in 96-well plates and incubated, at 37°C and 5% CO_2_, with twofold serial dilutions of EOMS (2 mg/mL–16 *μ*g/mL in DMEM) for 1 or 48 h (the cells incubated for 1 h were washed with PBS and further incubated in fresh medium). After 48 h incubation, HeLa cells were incubated with MTT reagent (5 mg/mL) for 4 h. Afterwards, the medium was removed and formazan crystals were dissolved with the MTT solubilisation solution (0.1 N HCl in isopropanol). The amount of formazan produced was detected by measuring the absorbance at 570 nm (ELISA reader). CC50 was defined as the product concentration required for reducing the cell viability by 50%.

### 2.4. Effect of EOMS towards Chlamydial EBs

Chlamydial EBs at a multiplicity of infection (MOI) of 0.05 IFU/cell were incubated in the presence or absence of EOMS for 30, 60, or 120 min at 37°C and 5% CO_2_. After the incubation period, the mixture containing chlamydial EBs and EOMS was diluted 1 : 100 in DMEM and used to infect HeLa cell monolayers grown in 24-well plates (2.0 × 10^5^ cells/well), as previously described. After 48 h of incubation at 37°C and 5% CO_2_ in cycloheximide-free medium, infected and treated cells were recovered to determine the infectivity yield and the IC_50_ value.

### 2.5. Effect of EOMS on* C. trachomatis* Replication

HeLa cell monolayers (2.0 × 10^5^ cells/well) grown in 24-well plates were infected with* C. trachomatis* (MOI 0.05) by centrifugation at 900 ×g at 37°C for 1 h. After removal of chlamydial inoculum, infected HeLa cells were incubated with twofold serial dilutions of EOMS prepared in culture medium. After 48 h of incubation at 37°C and 5% CO_2_ in cycloheximide-free medium, infected and treated cells were recovered to determine the infectivity yield and the IC_50_ value.

### 2.6. Effect of EOMS on Different Phases of Chlamydial Infection

EOMS was added during different phases of* C. trachomatis* infection as follows: (A) chlamydial EBs suspension was preincubated with EOMS for 2 h; (B) HeLa cells, grown in 24-well plates (2.0 × 10^5^ cells/well), were preincubated with EOMS for 2 h; (C) EOMS was added during* C. trachomatis* infection; and (D) EOMS was added during the 48 h postinfection period.

In all conditions, cells were incubated at 37°C and 5% CO_2_ in cycloheximide-free medium. After 48 h of incubation, infected and treated cells were recovered to determine the infectivity yield and the IC_50_ value.

### 2.7. Effect of EOMS in Combination with Erythromycin

HeLa cell monolayers (2.0 × 10^5^ cells/well) grown in 24-well plates were infected with* C. trachomatis* (MOI 0.05) by centrifugation at 900 ×g at 37°C for 1 h. After removal of chlamydial inoculum, infected HeLa cells were incubated with different concentrations of erythromycin (0.008–0.5 *μ*g/mL). After 48 h incubation at 37°C and 5% CO_2_ in cycloheximide-free medium, infected and treated cells were recovered and used to determine the infectivity yield and the IC_50_ value.

The combined effect between EOMS and erythromycin was assessed after infection of HeLa cells with* C. trachomatis* (MOI 0.05) as above described. Following removal of chlamydial inoculum, HeLa cells monolayers were treated with EOMS and subinhibitory doses of erythromycin. After 48 h of incubation at 37°C and 5% CO_2_ in cycloheximide-free medium, infected and treated cells were recovered to determine the infectivity yield and the IC_50_ value.

### 2.8. Assessment of Infectivity Yield

Infectivity yield was determined by the development of inclusions after passage to fresh HeLa cell monolayers. Briefly, cell monolayers were disrupted and repassed onto fresh HeLa cell monolayers grown on glass coverslips in 24 well-plates. After 48 h of incubation at 37°C and 5% CO_2_, the total number of* C. trachomatis* IFUs was counted by immunofluorescence assay.

The product concentration required to reduce chlamydial infectivity yield by 50% was defined as IC_50_.

### 2.9. Statistical Analysis

All values are expressed as mean ± standard deviation (SD) of three replicates from three independent experiments. Comparison of means was performed by using a two-tailed *t*-test for independent samples. A value of *P* < 0.05 was considered statistically significant.

## 3. Results

### 3.1. Cytotoxicity of EOMS

In order to assess the cytotoxic effect of EOMS, HeLa cell monolayers were incubated with increasing concentrations of essential oil for 1 h or 48 h and, then, cell viability was measured by the MTT assay.

**Figure 1:**
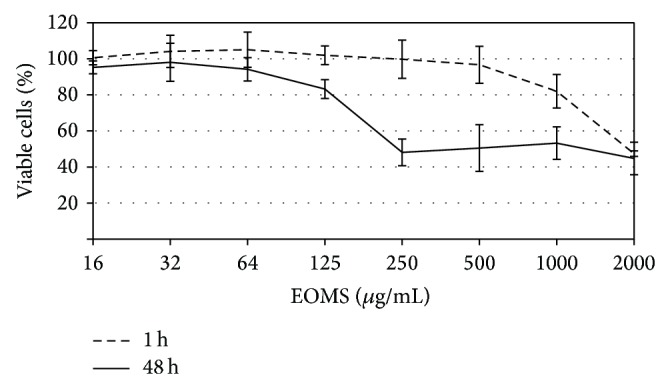
Cytotoxic effect of EOMS on HeLa cells. HeLa cell monolayers were treated with different concentrations of EOMS for 1 h or 48 h and, then, cell viability was determined by MTT assay.

No significant cytotoxicity was observed following 1 h exposure of EOMS up to 500 *μ*g/mL (Figure 1). The CC50 of EOMS was 1000 *μ*g/mL.

Following 48 h exposure of EOMS, a cytotoxic effect to HeLa cell monolayers was observed at a concentration of 125 *μ*g/mL (CC50).

### 3.2. Effect of EOMS on Chlamydial EBs

**Figure 2:**
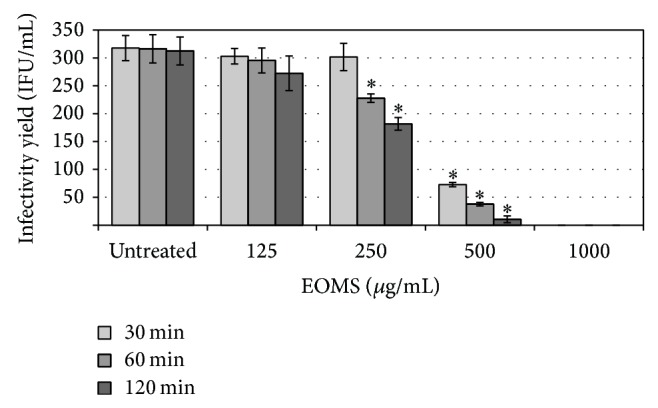
Inhibition of* C. trachomatis* EBs by EOMS treatment. Chlamydial EBs suspensions were treated with different concentrations of EOMS for 30, 60, or 120 min and then the mixture was used to infect HeLa cell monolayers. After 48 h of incubation, treated and untreated HeLa cell monolayers were recovered to determine the chlamydial infectivity yield. ^*^ *P* < 0.05 versus untreated cells.

In order to determine whether EOMS was active against chlamydial EBs, twofold serial dilutions of essential oil (from 1000 *μ*g/mL to 125 *μ*g/mL) were incubated with the EBs suspension for different periods. As shown in Figure 2, exposure of chlamydial EBs to concentrations of EOMS greater than 125 *μ*g/mL resulted in the inhibition of chlamydial infection as evidenced by a significant reduction in infectivity yield after 60 min of treatment as compared to infected cells alone (*P* = 0.04). A more marked reduction in infectivity yield was observed when the chlamydial EBs suspension was treated with 500 *μ*g/mL EOMS for 30, 60, or 120 min as compared to infected cells alone (*P* = 0.004; *P* = 0.003; and *P* = 0.003); a higher reduction in infectivity yield was observed in infected and treated cells for 60 or 120 min as compared to infected and treated cells for 30 min (30 min versus 60 min, *P* = 0.01; 30 min versus 120 min, *P* = 0.008). Again, a higher reduction in infectivity yield was observed in infected and treated cells for 120 min as compared to infected and treated cells for 60 min (*P* = 0.02).

Chlamydial EBs were completely inactivated following exposure to 1000 *μ*g/mL EOMS for 30 min (Figure 2).

The IC_50_ value for EOMS was 500 *μ*g/mL after 30 min of incubation whereas it was reduced to 250 *μ*g/mL after 60 and 120 min of incubation.

### 3.3. Effect of EOMS on* C. trachomatis* Replication

**Figure 3:**
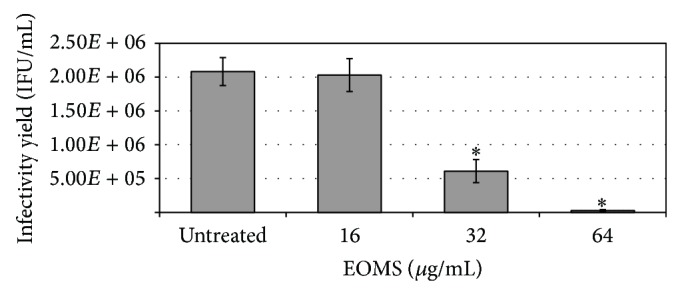
Inhibition of* C. trachomatis* replication by EOMS treatment. HeLa cell monolayers were infected with* C. trachomatis* (MOI 0.05) and then treated with different concentrations of EOMS. After 48 h of incubation, treated and untreated HeLa cell monolayers were recovered to determine the chlamydial infectivity yield. ^*^ *P* < 0.05 versus untreated cells.

The effect of EOMS on* C. trachomatis* replication was evaluated by treating infected cells with increasing concentrations of essential oil. EOMS at a concentration of 32 *μ*g/mL inhibited the chlamydial replication as evidenced by a significant reduction in infectivity yield observed in infected and treated cells as compared to infected cells alone (*P* = 0.002). EOMS was more effective in inhibiting* C. trachomatis* at a concentration of 64 *μ*g/mL (*P* = 0.004). A higher inhibitory effect of EOMS on* C. trachomatis* was observed in cells treated with 64 *μ*g/mL as compared to cells treated with 32 *μ*g/mL (*P* = 0.025) (Figure 3). The IC_50_ value of EOMS was 32 *μ*g/mL.

**Figure 4:**
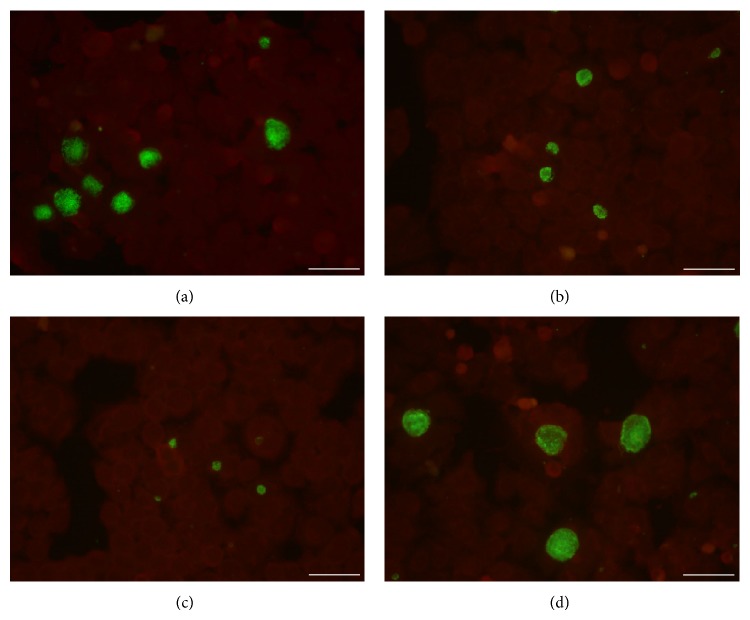
Immunohistological staining of* C. trachomatis* infected cell monolayers in the presence or absence of EOMS. HeLa cell monolayers were infected with* C. trachomatis* (MOI 0.05) and incubated in the presence ((a) 16 *μ*g/mL; (b) 32 *μ*g/mL; and (c) 64 *μ*g/mL) or absence of EOMS (d). After 48 h of incubation, HeLa cell monolayers were fixed, stained, and visualised by fluorescence microscopy (400x magnification). Bars: 50 *μ*m.

Interestingly, the sizes of individual chlamydial inclusions in cells infected and treated with EOMS were much smaller than those of infected cells alone, although it was not quantified. The effect of EOMS in chlamydial inclusion size reduction was dose dependent. Indeed, chlamydial inclusions from cells treated with 64 *μ*g/mL were much smaller as compared to those from cells treated with 32 *μ*g/mL (Figure 4).

**Figure 5:**
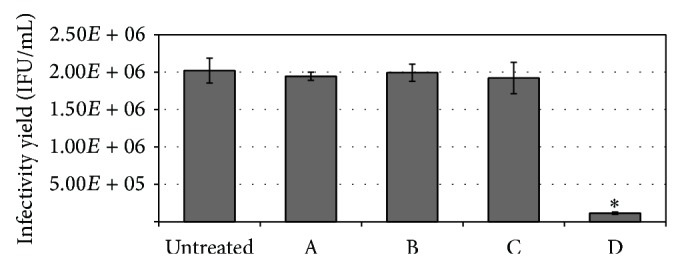
Effects of EOMS (64 *μ*g/mL) on different phases of* C. trachomatis* infection. (A) chlamydial EBs pretreatment with EOMS for 2 h. (B) HeLa cell monolayer pretreatment with EOMS for 2 h. (C) Addition of EOMS during chlamydial infection. (D) Addition of EOMS during postinfection period. After 48 h of incubation, treated and untreated HeLa cell monolayers were recovered to determine the chlamydial infectivity yield. ^*^ *P* < 0.05 versus untreated cells.

Based on the best inhibitory effect towards* C. trachomatis* and the absence of cytotoxicity of EOMS at 64 *μ*g/mL we used this concentration to determine more specifically which phase of chlamydial infection was impaired by the essential oil. The treatment conditions included pretreatment of chlamydial EBs suspension for 2 h, the pretreatment of host cells for 2 h, the coincubation of EOMS and* C. trachomatis* with host cells, and the addition of EOMS during the* C. trachomatis* postinfection period of 48 h. As shown in Figure 5, a significant reduction in infectivity yield was observed when EOMS was added during the 48 h postinfection phase as compared to infected cells alone (*P* = 0.002).

### 3.4. Effect of EOMS in Combination with Erythromycin

We first determined the activity of erythromycin towards* C. trachomatis* by treating infected cells with different concentrations of antibiotic (0.008–0.5 *μ*g/mL) for 48 h. The IC_50_ of the erythromycin was 0.030 *μ*g/mL, and at a concentration of 0.064 *μ*g/mL the chlamydial replication was completely inhibited.

Next, we evaluated the effects of EOMS in combination with erythromycin towards* C. trachomatis* by treating infected cells with 64 *μ*g/mL EOMS and subinhibitory doses of erythromycin. Erythromycin in combination with EOMS completely inhibited* C. trachomatis* replication at a concentration of 0.016 *μ*g/mL with IC_50_ of 0.007 *μ*g/mL.

## 4. Discussion

*C. trachomatis* is a significant public health problem because of the impact of asymptomatic infections on reproductive outcomes, the transmission of other sexually acquired agents, and the severe sequelae associated with the persistent forms. Indeed, in women, up to 40% of* C. trachomatis* untreated infections progress to serious complications [2], and there is also the evidence that an existing chlamydial genital infection may increase the risk of acquiring HSV-2 [25] and HIV [2, 3]. In particular, in the presence of sexually transmitted diseases including* C. trachomatis* infection the risk of HIV transmission may increase by three- to tenfold [26].

Lastly,* C. trachomatis* persistent forms are of particular clinical importance considering that they seem to be involved in the pathogenesis of adverse reproductive consequences of* C. trachomatis* infection since they may act as chronic stimuli leading to chronic inflammatory state and subsequent tissue damage [27].

In our study, we evaluated the activity of EOMS towards* C. trachomatis* in view of its safety and beneficial effect in topical treatment of vaginal candidiasis [28]. In particular, we investigated the aggressive LGV serovar of* C. trachomatis* known for its increasing incidence in Western Europe and North America [29, 30] and for its association with increased risk of HIV acquisition in several underdeveloped countries [31, 32].

Our results demonstrated that EOMS was not cytotoxic to epithelial cell line as was also evidenced in other studies [23, 28] and it was effective against* C. trachomatis*. Specifically, EOMS had a direct effect on chlamydial infectious EBs as shown by the significant reduction in infectivity yield observed after treatment of EBs with essential oil (IC_50_: 500 *μ*g/mL, 30 min). It is likely that EOMS inactivates EBs by disrupting lipid bilayers or by interfering with chlamydial components, which are essential for the entry into host cells, a fundamental stage in chlamydial infection cycle and pathogenesis.

In addition to inhibiting chlamydial EBs, EOMS was also effective after entry of* C. trachomatis* into host cells as shown by the significant reduction in infectivity yield after 48 h of treatment (IC_50_: 32 *μ*g/mL). Such effect was also demonstrated by the decreased size of individual chlamydial inclusions observed in infected and treated cells as compared to infected cells alone. The inhibitory effect of EOMS on chlamydial replication was further corroborated by the observation that early stages of* C. trachomatis* developmental cycle including the adhesion and the entry into host cell were not impaired by EOMS treatment.

As known, the management and control of* C. trachomatis* infection become increasingly challenging. Currently, erythromycin and azithromycin are recommended for treating* C. trachomatis* infections although these drugs have been shown to have side effects [33, 34]. In this regard, a particularly interesting finding of our study was the effectiveness of EOMS in combination with erythromycin against* C. trachomatis* with a considerable fourfold reduction in the minimum effective dose of antibiotic. The combination of EOMS antibiotic has shed light on a novel approach in controlling* C. trachomatis* infection and in decreasing the adverse effects of antibiotics. Limiting the effective dose of antibiotic reduces also the costs in financial terms since the combination of existing drugs require less costs than developing new drugs.

Thus, EOMS is effective towards* C. trachomatis* by several ways. First, EOMS inactivates* C. trachomatis* EBs supposedly preventing the entry in host cell, key phase for establishing the infection. Second, EOMS inhibits* C. trachomatis* replication reducing the infectivity and hence the progression of infection. Third, EOMS in combination with erythromycin inhibits* C. trachomatis* replication reducing the minimum effective dose of the antibiotic.

## 5. Conclusion

The present study revealed, for the first time, that EOMS was effective towards* C. trachomatis* by inactivating infectious elementary bodies and inhibiting chlamydial replication. These findings suggest that EOMS treatment may represent a preventative strategy for reducing* C. trachomatis* transmission in the population, the number of new chlamydial infections, and the risk of developing severe sequelae, even if further studies on the activity of EOMS towards vaginal bacterial flora and other sexually acquired agents are needed.
